# Enteral feeding advancement and growth until 5 years in extremely preterm infants

**DOI:** 10.1186/s12887-021-02878-8

**Published:** 2021-09-23

**Authors:** Cornelia Wiechers, Jan-Niklas Doll, Christoph Maas, Kerstin Gründler, Katja Büchner, Christian F. Poets, Axel R. Franz

**Affiliations:** 1 Department of Neonatology, University Children’s Hospital, Eberhard Karls University, Tuebingen, Calwerstr. 7, 72076 Tuebingen, Germany; 2 Center for Pediatric Clinical Studies, University Children’s Hospital, Eberhard Karls University, Tuebingen, Germany

**Keywords:** Infant, Neonatal, Premature, Nutrition, Enteral feeding, Growth outcome

## Abstract

**Background:**

In-utero weight gain can be achieved in very preterm infants through rapid advancement of enteral feeds without increasing risk of necrotizing enterocolitis. There are concerns, however, that such rapid weight gain may lead to an increased childhood adiposity risk, although long-term data are sparse.

**Design:**

This retrospective observational study included two well-characterized cohorts comprising 145 infants born at < 28 weeks or with < 1000 g birth weight. We investigated associations between advancing enteral feeding volumes in daily increments of 15–20 ml/kg (Cohort 1, *n* = 84, born in 2006/2007) vs. 25–30 ml/kg (Cohort 2, *n* = 61, born in 2010) and growth up to 5 years of age.

**Results:**

There was no significant difference in anthropometric parameters post discharge to 5 years between both cohorts. Standard deviation score (SDS) weight and SDS BMI at the age of 5 years remained lower than in the reference population. SDS weight decreased from discharge to about 10–12 months postnatal age and returned to birth values by age 5 years. There was a catch-up for SDS length/height from discharge to 5 years; SDS head circumference decreased from birth to 5 years. Multiple regression analyses revealed that for all anthropometric parameters SDS at birth was the most important predictor for SDS at 5 years. Early parenteral protein intake may be another important factor, at least for head growth.

**Conclusions:**

Growth was similar in both cohorts without benefit from more accelerated feeding advancement in cohort 2. In both cohorts, early enteral nutrition was associated with in-hospital weight gain as in utero, a drop in weight SDS post discharge and catch-up to birth SDS until age 5 years, remaining below the reference population. Length showed catch-up form discharge to 5 years, whereas head circumference progressively deviated from the reference population. Increased parenteral protein supplementation may be needed to accompany early enteral feeding advancements.

## Background

Intensive nutritional support for preterm infants improves growth and thereby potentially neurodevelopment [1–3]. Therefore, most pediatric societies recommend that postnatal growth in the NICU should match that observed in utero [4].

Reaching this goal may require intensified enteral nutrition and faster feeding advancements, thereby also reducing time with intravenous access and parenteral nutrition, and the risk of sepsis and cholestasis [5]. The optimal rate of enteral feeding advancements in preterm infants is yet unknown, but available data suggest that advancing enteral feeding volumes at daily increments of 30–40 ml/kg does not increase the risk of necrotizing enterocolitis (NEC) or death even in very preterm or growth-restricted infants [5–8].

Especially after rapid advancement of enteral feeding volumes, there is concern that preterm infants’ growth could be impaired by the variability in protein and energy content of human milk or insufficient enteral macronutrient absorption immediately after birth [9]. In a recent large randomized trial involving 2804 preterm infants, no difference could be found in developmentally intact survival at 24 months with a strategy of advancing milk feeding volumes in daily increments of 30 ml/kg as compared to 18 ml/kg [7]. However, long-term data on growth in later childhood and metabolic outcome of premature infants after accelerated enteral feeding advancement are sparse.

We studied the effect of accelerated enteral feeding advancements in infants born at < 32 wk. gestation or with birth weights < 1500 g [8]. Time to full enteral feeds was significantly shorter in the cohort with faster advancement of enteral feeding volumes (6 vs. 8 days), while NEC rates were similar (3.3 vs. 2.7%). In contrast to published data reporting extrauterine growth restriction among most very preterm infants [10–14], both study cohorts continued to grow along their intrauterine percentiles. Better head circumference growth in the cohort with slower enteral feeding advancement was associated with higher parenteral protein administration in the first postnatal week [8].

We now aimed to investigate the effects of accelerated enteral feeding advancements on growth between hospital discharge and age 5 years in extremely preterm or extremely low birth weight infants previously described including details on postnatal nutrition [8].

## Methods

### Participants/ study population

This was a retrospective, single center observational study of extremely premature infants (< 28 0/7 weeks gestation age or with birth weight < 1000 g) born between January 2006 and December 2007 or between January 2010 and December 2010 at Tuebingen University Women’s and Children’s Hospital, Germany, limited to infants who survived to discharge home. In cohort 1, eight infants died before and three after discharge and in cohort 2, seven infants died before and no infant died after discharge. Infants who died were not included in this study. The initial course of both cohorts has been extensively described [8]. Infants with congenital malformations of the gastrointestinal tract, death before age 5 years or transfer to another hospital before achieving full enteral feeds were excluded.

### Data collection

Clinical and anthropometric data between birth and discharge were determined daily. Furthermore, exact nutritional intakes were evaluated daily for the first 28 days after birth. Children’s growth data between hospital discharge and age 5 years were collected during routine in-house follow-up and additionally by means of a parental questionnaire collecting data from the child’s routine developmental screening examinations designated by German law. The time intervals for these screening examinations are at 6 to 7 months of postnatal age (S1), 10 to 12 months (S2), 21 to 24 months (S3), 46–48 months (S4) and 60–64 months (S5).

### Ethics

The Institutional Review Board approved the study protocol and written informed parental consent was obtained (project number: 210/2018BO2).

### Enteral nutrition policy

The previously established feeding protocol was replaced by a revised version in 2009 to allow for an even faster advancement of enteral feeding volumes, changes to our feeding protocol are described in Table 1 and by Maas et al. [8]. According to the modified feeding protocol, preterm infants were divided into cohort 1 (2006–2007) and cohort 2 (2010).

**Table 1:** Differences in feeding protocols between cohorts

	Cohort 1	Cohort 2
Start of enteral feeds within 4 h of birth	10–15 ml/kg/d	20 ml/kg/d
Increase in daily enteral feeding volume	15–20 ml/kg/day	25–30 ml/kg/day
Cut-off for of gastric residual volume considered “normal” (in infants with unremarkable abdominal clinical status)	1–2 ml/kg	< 4 ml/kg
Start of breast milk fortification (FM85, Nestle, Frankfurt) at enteral feeding intake	≥150 ml/kg/d	≥100 ml/kg/d
Parenteral protein administration (day1)	2.5 g/kg/day	3.0 g/kg/d

Feeding own mother’s milk was encouraged; if no breast milk was available, formula was fed (Beba, Nestlé). In both cohorts, enteral feeds started within 4 h and parenteral nutrition immediately after birth. Full enteral feeds were defined as ≥140 ml/kg/d administered for 24 consecutive hours. To calculate macronutrient supply, we assumed a protein content in human milk of 1.4 g/100 ml and 67 kcal/100 ml.

### Calculation of standard deviation scores (SDS) for weight (SDS_W_), length (SDS_L_) and head circumference (SDS_HC_)

Anthroprometric parameters were computed using LMSgrowth (version 2.14; http://www.healthforallchildren.com/?product=lmsgrowth). The reference population was the British 1990 growth reference [15, 16] fitted by maximum penalized likelihood as described before [15].

### Statistical analyses

Data are presented as mean (standard deviation, SD) if normally distributed, or as median and interquartile range if not. In case that within a table a minority of parameters were normally distributed, the data are still presented as median (interquartile range) to improve clarity of presentation. Comparisons between groups were performed using a two-sided t-test or ANOVA and post hoc Tukey’s multiple comparison test for normally distributed variables or Wilcoxon test in non-normally distributed data and Chi-square test for categorical outcomes. Multiple linear regression analysis was performed to identify important influencing factors on SDS_W_, SDS_L_ and SDS_HC_ at 5 years including study cohort, sex, gestational age and SDS at birth, and based on our previous analysis [8] cumulative parenteral protein intake until 28d, as independent variables. Despite a difference in the proportion of multiple births between cohorts, we did not include multiple births into this analysis because the main effect being a multiple should be on birth weight (not on postnatal growth) and for each anthropometric measure the corresponding SDS at birth was included. Analyses were performed with GraphPad Prism® 8.1.0 (GraphPad Software, San Diego, CA, USA) and SAS 9.4, the level of significance was *p* < 0.05.

## Results

### Participants

A total of 163 premature infants with gestational age < 28 weeks or birth weight < 1000 g were admitted to our hospital during the two study periods; 18 (11.0%) died before age 5 years and were thus excluded from analysis (11/95 (11.6%) in cohort 1, 7/68 (10.3%) in cohort 2). No infant had a severe congenital malformation of the gastrointestinal tract. Demographic data of the study population are shown in Table 2. Complete anthropometric data were available for all 145 infants at birth and discharge, the proportion of infants with documented growth data at the time of S1 (at 6–7 months) was 90% and decreased in the following periods: S2: 86%, S3: 82%, S4: 69%, S5: 65% (Tables 3 and 4).

**Table 2:** Characteristics of study cohorts

		Cohort 1 ***n*** = 84	Cohort 2 ***n*** = 61
Female	n (%)	48 (57%)	38 (62%)^e^
Gestational age at birth (weeks)	Median (Q1, Q3)	26.1 (24.9–27.6)	27.0 (26.0–27.7**)**^e^
Birth weight (g)	Median (Q1, Q3)	740 (597–932)	785 (620–980)
Birth length (cm)	Median (Q1, Q3)	33 (31–35)	33 (31–35)
Birth head circumference (cm)	Median (Q1, Q3)	22.5 (22.0–25.0)	24.0 (22.5–25.7)
Infants from multiple pregnancies	n (%)	22 (26.2%)	36 (59.0%)^e^
Twins	n (%)	18 (21.4%)	28 (45.9%)
Triplets	n (%)	4 (4.8%)	8 (13.1%)
Length of hospital stay (days)	Median (Q1, Q3)	89 (64–109)	77 (55–102)
Postmenstrual age at discharge (weeks)	Median (Q1, Q3)	39.3 (37.2–41.0)	38.4 (36.0–40.7)
**Neonatal morbidities**			
Necrotizing enterocolitis^b^	n (%)	2 (2.4%)	2 (3.3%)
Focal intestinal perforation	n (%)	5 (6.0%)	2 (3.3%)
Intraventricular hemorrhage ≥III	n (%)	7 (8.3%)	2 (3.3%)
Periventricular leukomalacia	n (%)	1 (1.2%)	3 (4.9%)
Bronchopulmonary dysplasia^a^	n (%)	21 (25%)	14 (23%)
Retinopathy of Prematurity requiring therapy	n (%)	8 (9.5%)	2 (3.2%)
**Total time with**^**c**^			
Peripheral venous line (days)	Median (Q1, Q3)	11 (7–19)	9 (5–15)^e^
Central venous line (days)	Median (Q1, Q3)	6 (0–14)	0 (0–6)^e^
Antibiotic treatment (days)	Median (Q1, Q3)	10 (5–22)	9 (5–17)
**Nutrition**			
Day of life when full enteral feeds were attained	Median (Q1, Q3)	9 (8–11)	7 (6–9)^e^
**Cumulative protein intake (g/kg)**			
First postnatal week	Median	25.5 (23.4–27.0)	24.2 (22.3–27.3)
First postnatal 28 days	(Q1, Q3)	104.6 (99.8–108.1)	109.0 (103.1–115.0)^e^
**Cumulative parenteral protein intake (g/kg)**			
First postnatal week	Median (Q1, Q3)	17.9 (15.7–20.0)	10.7 (8.3–15.0)^e^
First postnatal 28 days	Median (Q1, Q3)	22.8 (17.1–30.3)	12.8 (8.3–22.7)^e^
**Cumulative energy intake (kcal/kg)**			
First postnatal week	Median (Q1, Q3)	592 (529–625)	653 (577–721)^e^
First postnatal 28 days	Median (Q1, Q3)	3358 (3109–3490)	3442 (3266–3673)^e^
**Nutrition at discharge**			
Mother’s own milk^d^ 100%	n (%)	25 (30%)	32 (53%)^e^
Mother’s own milk^d^ & Formula	n (%)	18 (21%)	14 (23%)
Formula 100%	n (%)	41 (49%)	15 (25%)^e^
**Nutrition at 3 months follow up**		*n* = 67	*n* = 54
Mother’s own milk^d^ 100%	n (%)	6 (9%)	11 (20%)
Mother’s own milk^d^ & Formula	n (%)	10 (15%)	6 (11%)
Formula 100%	n (%)	51 (76%)	37 (69%)
**Duration of expressed breast milk/breastfeeding** (months)		*n* = 39	*n* = 45
Based on questionnaire	Median (Q1, Q3)	5 (3–9)	5 (4–9)

Cohort 1 (2006–2007): enteral feeds were initiated on day 1 with 10–15 ml/kg/day and advanced by 15–20 ml/kg/day

Cohort 2 (2010): enteral feeds were initiated on day 1 with 20 ml/kg/day and advanced by 25–30 ml/kg/day

^a^ Moderate or severe BPD according to consensus definition based on oxygen supplementation or positive pressure respiratory support at 36 weeks PMA

^b^ ≥ IIa according to Bell criteria

^c^ During the entire inpatient stay

^d^ Supplemented with human milk fortifier

^e^ Significantly different between the two cohorts (*p* < 0.05 by Chi-square and Mann-Whitney-Test, respectively)

**Table 3:** Weight, SDS weight, SDS length/height, SDS head circumference and SDS BMI from birth to age five years

Time	Cohort 1						Cohort 2					
	n	Weight (kg)	SDS weight	SDS length/height	SDS head circumference	SDS BMI	n	Weight (kg)	SDS weight	SDS length/height	SDS head circumference	SDS BMI
**Birth**	84	0.740 (0.597–0.932)	− 1.17 (− 2.09 - -0.23)	N/A	− 1.39 (− 2.04 - -0.50)	N/A	61	0.785 (0.620–0.980)	− 1.40 (− 2.24 - -0.61)	N/A	− 1.16 (− 2.06 - -0.52)	N/A
**Discharge**	84	2.692 (2.370–3.044)	− 1.24 (− 2.01 - -0.69)	− 2.24 (− 3.17 - -1.58)	− 0.72 (− 1.55–0.09)	0.27 (− 0.38–1.02)	61	2.530 * (2.028–2.858)	− 1.42 (− 2.35 - -0.68)	−2.65 (− 3.39 - -1.75)	−1.17* (− 2.06 - -0.76)	0.18 (− 0.41–1.01)
**S1** (6–7 months)	74	5.22 (4.74–5.85)	− 1.47 (− 2.21 - -0.77)	−1.31 (− 1.85 - -0.41)	−0.81 (− 1.7 - -0.10)	−1.09 (− 1.72 - -0.36)	56	5.02 (4.56–5.80)	−1.84 (− 2.54 - -0.81)	−1,35 (− 2.25 - -0.26)	−1.19 (− 2.02 - -0.49)	−1.52 (− 2.16 - -0.46)
**S2** (10–12 months)	74	7.31 (6.66–8.21)	−1.68 (− 2.62 - -1.00)	−0.75 (− 1.26–0.18)	− 1.40 (− 2.22 - -0.85)	−1.67 (− 2.8 - -0.98)	52	7.09 (6.29–7.98)	−2.07 (− 3.17 - -0.99)	−0.61 (− 1.47–0.25)	−1.69 (− 2.75 - -1.13)	−2.06 (− 2.92 - -1.16)
**S3** (21–24 months)	74	10.00 (9.36–10.92)	−1.46 (− 2.16 - -0.77)	−0.74 (− 1.35 - -0.09)	−1.67 (− 2.46 - -0.75)	−1.44 (− 2.36 - -0.64)	45	9.53 (8.62–10.50)	−1.79 (− 3.09 - -1.13)	−0.65 (− 1.84 - -0.06)	−2.12 (− 2.90 - -1.37)	−1.91 (− 2,77 - -0.77)
**S4** (46–48 months)	58	13.65 (12.42–15.00)	−1.30 (− 2.11 - -0.35)	−0.32 (− 0.92–0.32)	− 1.89 (− 2.52 - -1.25)	−1.48 (− 2.23 - -0.7)	42	13.50 (12.00–15.00)	−1.51 (− 2.35 - − 0.39)	-0.39 (− 1.14–0.07)	−2.09 (− 3.23 - -1.31)	−1.76 (− 2.18 - -0.79)
**S5** (60–64 months)	51	16.00 (14.35–18.00)	−1.08 (− 1.91 - -0.06)	− 0.16 (− 1.02–0.28)	−1.78 (− 2.66 - -1.17)	−1.14 (− 1.87 - -0.46)	43	15.65 (13.80–17.57)	−1.51 (− 2.36 - -0.35)	− 0.65 (− 1.58–0.13)	−2.17 (− 3.04 - -1.38)	−1.27 (− 2.10 - -0.71)

All data presented as median (Q1, Q3), N/A not available (SDS length and SDS BMI could not be computed before term)

Abbreviations: Body mass index (BMI), Standard deviation score (SDS)

*Significantly different between the two cohorts (*p* < 0.05; Mann-Whitney-Test)

Child’s routine developmental screening examinations: S1: 6 to 7 months after birth, S2: 10 to 12 months after birth, S3: 21 to 24 months after birth, S4: 46–48 months after birth, S5: 60–64 months after birth

**Table 4:** SDS-Difference from birth to discharge and to age five years

Difference of SDS	Cohort 1	Cohort 2
**SDS**_**discharge**_**– SDS**_**birth**_		
Weight	−0.11 (− 0.83–0.37)	−0.15 (− 0.61–0.35)
Head circumference	0.48 (− 0.32–1.30)	− 0.20* (− 0.71–0.75)
Length	N/A	N/A
BMI	N/A	N/A
**SDS**_**S5**_**– SDS**_**birth**_		
Weight	0.1 (− 0.6–1.0)	0.0 (− 1.3–0.7)
Head circumference	−0.9 (− 1.7–0.0)	−0.8 (− 1.7 – − 0.1)
Length/height	N/A	N/A
BMI	N/A	N/A
**SDS**_**S5**_**–SDS**_**discharge**_		
Weight	0.3 (−0.5**–**1.2)	0.0 (− 0.6–0.7)
Head circumference	− 1.3 (− 2.1 – − 0.5)	−1.2 (− 1.6–0.1)
Length/height	2.0 (1.0–2.8)	1.8 (1.1–2.4)
BMI	−1.5 (− 2.2 – − 0.6)	−1.5 (− 2.0 – − 0.7)

All data presented as median (Q1, Q3).

*Significantly different between the two cohorts (*p* < 0.05 by Mann-Whitney-Test)

N/A not available (SDS for length and for BMI could not be computed at birth before term).

Abbreviations: Body mass index (*BMI*), Standard deviation score (*SDS*), SDS S5: 60–64 months after birth (SDS_S5_)

In Cohort 1, the proportion of multiple births and gestational age at birth were lower than in cohort 2 (26% vs. 59%, *p* < 0.0001, and 26.1 vs. 27.0 wk., *p* = 0.037, respectively). Full enteral feeds were established significantly later in cohort 1 (median 9th vs. 7th postnatal day, *p* < 0.0001; Table 1). Cumulative energy and cumulative protein intake were lower whereas cumulative parenteral protein intake was higher in Cohort 1 than in Cohort 2 (all *p* < 0.01, Table 2). There was no difference in the length of hospital stay (Table 2). Median duration with peripherally inserted central venous access and with peripheral venous access were both shorter in cohort 2 (*p* = 0.006 and *p* = 0.044; Table 2).

**Fig. 1:**
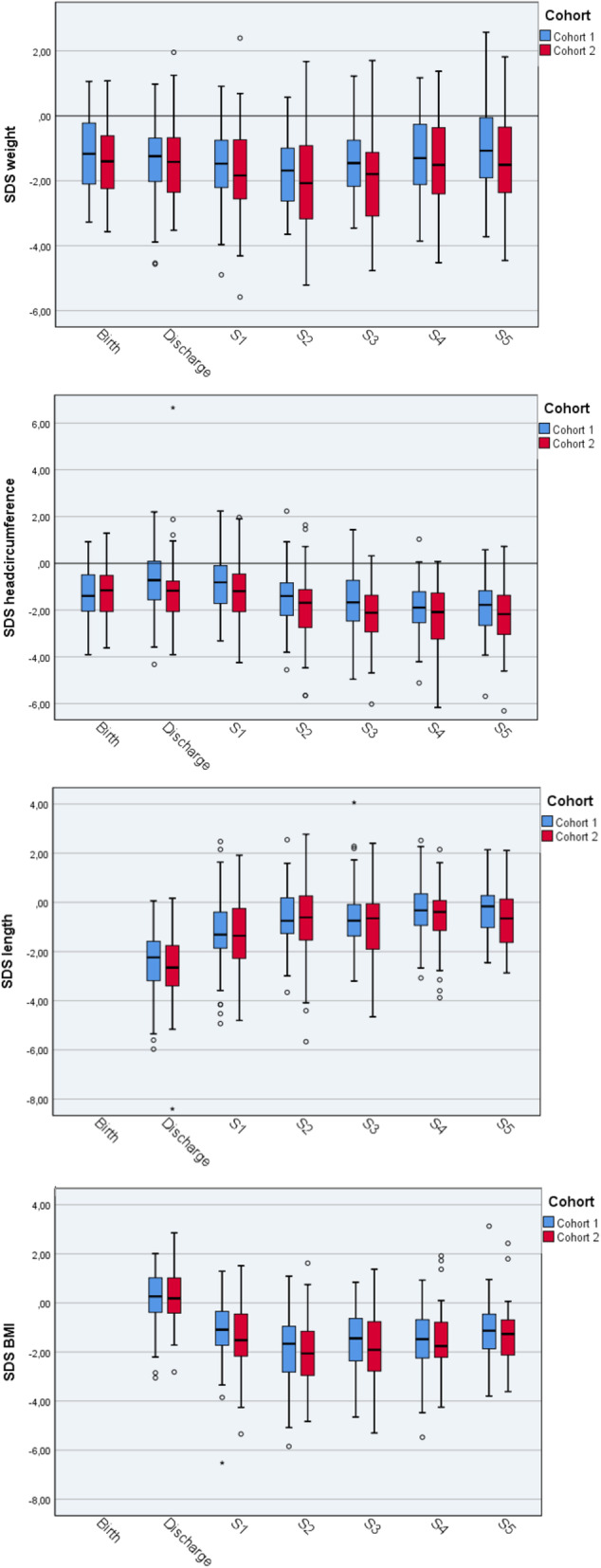
SDS for weight, head circumference, length/height and BMI from birth to age five years (S5)

Median SDS_W Birth_ was similar with − 1.17 (− 2.09 - -0.23) in cohort 1 and − 1.40 (− 2.24 - -0.61) in cohort 2. 23/84 infants (27%) in cohort 1 and 20/61 (33%) in cohort 2 were born small for gestational age with a weight less than 2 standard deviations below the mean (Table 3, Fig. 1). Differences in SDS from birth to discharge and to age 5 years, respectively, were close to zero and not different between cohorts (Table 4). However, SDS_W_ decreased post discharge with a nadir for SDS_W_ at S2 (10–12 months after birth or 6–8 months post discharge) and a recovery towards birth values by 5 years. By contrast, there was a steady decline in SDS_HC_ and a continuous catch-up of SDS_L_ from discharge to age 5 years (Fig. 1).

On multiple linear regression analysis, SDS_W_, SDS_L_, SDS_HC_ at birth were the most important influencing factors for SDS_W_, SDS_L_, SDS_HC_ at 5 years. However, r^2^ was only 0.16, 0.32 and 0.21 respectively (Table 5). Cumulative parenteral protein intake at d1-d28 was a significant predictor of SDS_HC_ at 5 years.

**Table 5:** Potential influencing factors for SDS at age 5 years by multiple linear regression

	Influence on SDS_**W**_		Influence on SDS_**HC**_		Influence on SDS_**L**_	
	*r*^*2*^ = 0.1573		*r*^*2*^ = 0.3219		*r*^*2*^ = 0.2070	
	Estimate	*p*-value	Estimate	*p*-value	Estimate	*p*-value
Intercept	−5.751	0.0322	−0.5291	ns	0.4486	ns
Gestational age at birth	0.1915	ns	−0.01476	ns	−0.01206	ns
SDS at birth ^a^ / at discharge ^b^	0.4286	**0.0041**	0.4930	**0.0058**	0.3374	**0.0004**
Gender	0.3009	ns	0.7060	ns	−0.09560	ns
Cohort1/2	−0.3275	ns	−0.5057	ns	−0.2198	ns
Cumulative parenteral protein intake d1-d28	−0.0004704	ns	−0.02922	**0.0236**	0.01227	ns

Abbreviations: not significant (*ns*), Standard deviation score (*SDS*)

^a^ For weight and head circumference; ^b^ for length/height

## Discussion

The aims of this study were to analyze and describe growth in extremely preterm infants during the first 5 years after birth following accelerated enteral feeding advancement and in-hospital weight gain as in utero, and to investigate factors potentially influencing anthropometric data until 5 years of age. Median postnatal age at full feeds (defined as ≥140 ml/kg/d administered for 24 consecutive hours) was 9 days and 7 days in these extremely preterm or extremely low birth weight infants.

Overall, no differences in growth pattern up to 5 years of age were found between the two cohorts with different feeding regimens. Compared to the reference population, the preterm infants studied here had a significantly lower SDS_W_, SDS_L_ and SDS_HC_ at birth. There were no differences in SDS_W_ between the two cohorts throughout the observational period. In Cohort 1, with slower daily feeding increments and higher parenteral protein intake during the first postnatal week, a better early postnatal head circumference growth was observed until discharge [8]. At age 5 years, this difference in head circumference and in change in SDS_HC_ was no longer evident. Nevertheless, cumulative parenteral protein intake after birth remained significantly associated with SDS_HC_ at 5 years in multiple linear regression analysis and SDS_HC_ progressively decreased up to age 5 years. As a consequence of this finding we have increased parenteral protein supplementation during the postnatal transition to full enteral feeds, but additional nutritional interventions (e.g., supplementation with choline and polyunsaturated fatty acids) should also be considered [17–20]. Catch-up growth occurred only for SDS_L_ in both cohorts, showing values within the reference range at age 5 years.

A Swedish follow-up study of 83 extremely preterm infants came to similar results: weight showed a sharp decrease in z-scores from birth to 3 months’ corrected age, followed by catch-up growth up to age 11 years, but weight remained lower than in term-born controls (mean (SD) z score: at birth − 0.57 (0.98), at due date − 1.62 (0.76), at 3 months − 2.27 (1.38), at 5 years − 0.77 (1.35) and at 11 years − 0.15 (1.22)) [21]. Z-scores for height decreased up to 3 months corrected age (− 2.24 (1.32)), followed by catch-up growth up to 11 years (− 0.53 (1.08). Head circumference also showed no catch-up growth and remained significantly lower than in control participants at all ages, mean z-Score_HC_ at birth was − 1.30 (0.71) and − 1.11 (1.06) at 11 years of age.

Contrary to our and the Swedish findings, many studies reported that preterm infants with postnatal growth retardation up to discharge regain weight in the following months, with catch up to their peers by the time of reaching school age [10, 21].

As currently recommended [4], both study cohorts matched in-utero weight gain during their stay in the NICU with a delta-SDS_W_ from birth to discharge of close to zero; likewise the change in SDS_W_ from birth to age 5 years was close to zero. The SDS_BMI_ at age 5 years was significantly lower than at discharge and lower than that of the reference population, but again not different between cohorts. Presumably, there were no differences in growth pattern up to age 5 years between the two cohorts because time to establish full enteral feeds was similarly short and cumulative protein intake until d28 was similarly high and higher than in most previous studies reporting increased weight gain with increased protein intake [1, 11]. Recently published randomized controlled trials on the effect of increased protein supplementation on growth of very preterm infants have shown that increasing protein intake by 0.6 g/kg/d to an intake of 4.3 g/kg/d does not further promote growth until discharge, possibly due to a ceiling effect [22, 23].

In contrast to our findings, most studies report that preterm infants fail to achieve near in-utero weight gain during their NICU stay [10–12, 21, 24]. A number of studies demonstrated an association between impaired postnatal growth in preterm infants and neurodevelopmental disabilities, hence the importance of early and adequate nutrition in this period of rapid growth [1–3].

Beyond neurocognitive development, it has been suspected that disturbances in intrauterine growth can have long-term effects on adult health, e.g., an increased risk for hypertension and insulin resistance, likely based on mechanisms of fetal programming and epigenetics [25, 26]. However, it is unclear whether extrauterine growth restriction in preterm infants has similar effects as fetal growth restriction [24, 27, 28]. For example, a higher prevalence of insulin resistance and glucose intolerance and increased systolic blood pressure have been described in 163 very low birth weight (VLBW) infants at age 18–27 years compared to young adults who were born at term [24]. In that study, extrauterine/postnatal weight gain was poor and mean SDS change from birth to term − 1.4 ± 1.32, very different from our observations. In a randomized study including 50 VLBW infants, a significant improvement in postnatal growth without Z-score loss from birth to 36 weeks corrected age was found with increased compared to standard nutrient supply [29]. In that study, adiponectin concentrations at 5 months corrected age were higher with increased nutrient supply and this correlated positively with early weight gain, where increased adiponectin levels are generally associated with a lower risk of obesity and metabolic syndrome in children [29, 30]. Early nutrition and growth may therefore influence metabolic markers in infancy and early childhood. The long-term metabolic effects of an in-hospital growth pattern mimicking intrauterine growth on cardiovascular and metabolic health require further study.

Limitations of our study include its retrospective and single-center design and up to 35% loss to follow-up until age 5 years. Potentially, the latter could introduce attrition bias and thus affect the validity of our observations, however, mean GA at birth of those followed to age 5 years and of the initial cohorts were similar. For this study, we deliberately did not contact parents whose children had already died before age 5 years, consequently our data only apply to infants who survive to 5 years. Furthermore, this study is obviously limited to survivors, the growth data of the sickest preterm infants, who died before discharge, have not been included. Although this is a retrospective cohort study in two periods 3–4 years apart, there were no significant changes in management between the two cohorts described, apart from modifying the feeding protocol. Remarkably, however, in cohort 1 the proportion of multiple births and gestational age were lower than in cohort 2, although we did not find an explanation for this increase.

Strengths include our well-described nutritional regimen and detailed information on nutritional support during the first 28 postnatal days, and that both cohorts included an unselected population of extremely low birth weight (ELBW) infants with intensified and very early enteral nutrition resulting in an early postnatal growth along their intrauterine percentiles during their NICU stay.

## Conclusion

Both cohorts attained full enteral feeds much earlier than previously reported and weight gain as in utero was achieved. There was no significant difference in anthropometric parameters after discharge between both cohorts, but there was catch-up for SDS length/height from discharge to age 5 years. SDS weight and SDS BMI at age 5 years remained lower than in the reference population, SDS weight decreased from discharge to about 10–12 months and returned to birth values at age 5 years. SDS head circumference decreased from birth to 5 years, thus higher parenteral protein supplementation may be necessary during transition to full enteral feeds.

In summary, there is no evidence of an increased risk of obesity up to age 5 years following intensified nutritional support with rapid enteral feeding advancement and percentile-parallel weight gain during their NICU stay. Further follow up into adulthood is desirable.

## Abbreviations

bw: Birth weight
EF: Enteral feeding
BMI: Body mass index
ELBW: Extremely low birth weight (< 1000 g)
FIP: Focal intestinal perforation
N/A: Not available
NEC: Necrotizing enterocolitis
NICU: Neonatal intensive care unit
VLBW: Very low birth weight (< 1500 g)
SD: Standard deviation
SDS: Standard deviation score
SDS_w birth_: SDS weight at birth
SDS_w S5_: SDS weight five years of age
wk.: Week
